# Relationship of Religion with Suicidal Ideation, Suicide Plan, Suicide Attempt, and Suicide Death: A Meta-analysis

**DOI:** 10.34172/jrhs.2022.72

**Published:** 2021-10-31

**Authors:** Jalal Poorolajal, Mahmoud Goudarzi, Fatemeh Gohari-Ensaf, Nahid Darvishi

**Affiliations:** ^1^Department of Epidemiology, School of Public Health, Hamadan University of Medical Sciences, Hamadan, Iran; ^2^Modeling of Noncommunicable Diseases Research Center, Hamadan University of Medical Sciences, Hamadan, Iran; ^3^Department of Family Counseling, Sanandaj Branch, Islamic Azad University, Sanandaj, Iran; ^4^Department of Psychology, School of Human Sciences, Sanandaj Branch, Islamic Azad University, Sanandaj, Iran

**Keywords:** Suicide plan, Suicidal ideation, Attempted suicide, Completed suicide, Meta-analysis, Religion, Spirituality

## Abstract

**Background:** Suicide is a significant public health problem and one of the leading causes of death worldwide. The effect of religion on suicidal behaviors (i.e., ideation, plan, attempt, and death) is an important issue worthy of consideration.

**Methods:** Major electronic databases, including MEDLINE, Web of Science, and Scopus, were searched for the articles published until 26 April 2021. Reference lists were also screened. Observational studies addressing the associations between religion and suicidal behaviors were also examined. Between-study heterogeneity was investigated using the χ^2^, τ^2^, and I^2^ statistics. The probability of publication bias was explored using the Begg and Egger tests, as well as trim-and-fill analysis. The effect size was expressed as odds ratio (OR) with 95% confidence intervals (CIs) using a random-effects model.

**Results:** Out of 11389 identified studies, 63 articles were eligible, involving 8,053,697 participants. There was an inverse association between religion and suicidal ideation OR = 0.83 (95% CI: 0.78, 0.88; *P*<0.001), suicidal plan OR = 0.93 (95% CI: 0.83, 1.04; *P* = 0.200), suicide attempt OR = 0.84 (95% CI: 0.79, 0.89; *P*<0.001), and completed suicide OR = 0.31 (95% CI: 0.14, 0.72; *P* = 0.006). There was a no evidence of publication bias.

**Conclusions:** The results of this meta-analysis support the notion that religion can play a protective role against suicidal behaviors. Nonetheless, the effect of religion on suicidal behaviors varies across countries with different religions and cultures. Although this association does not necessarily imply causation, an awareness of the relationship between religion and suicide risk can be of great help in suicide prevention policies and programs.

## Background

 Suicide is one of the top 20 leading causes of death and premature mortality in people of all ages across the globe,^1,2^ the third major cause of death among people aged 15-44 years, and the second leading cause of death in 10-24 year-olds.^3^ The individuals who die due to suicide outnumber those who die in war. In fact, for every death caused by conflict, five deaths are caused by suicide.^4^ Based on the World Health Organization (WHO), around one million people die from suicide every year, resulting in a global mortality rate of 16 per 100 000, or one death every 40 seconds.^3^ These figures understate the problem since they do not include attempted suicides, which are up to 20 times more common than suicide deaths;^3^ moreover, many people who have suicidal thoughts never seek services.^5^

 Evidence suggests that there is no known single cause for suicide, rather it is a complicated event influenced by a variety of psychological, social, biological, cultural, and environmental factors.^3,6,7^ Epidemiological research has demonstrated that several behavioral factors, such as alcohol consumption,^8^ drug abuse,^9^ and smoking,^10^ have a role to play in suicide. Another factor that plays a pivotal role in one’s lifestyle, general health, and wellbeing is religion.^11^ Based on the Gallup surveys conducted in 114 countries in 2009, religion plays an essential role in the lives of numerous people around the world. About 84% of adults reported that religion was an essential part of their daily lives. In 10 nations and territories, at least 98% of people claimed that religion was significant in their daily lives.^12^ Another poll conducted by Gallup International in 2012 involving 50 000 people selected from 57 countries across the world in five continents revealed that 59%, 23%, and 13% of participants considered themselves to be religious, non-religious, and convinced atheists, respectively.^13^

 The relationship between religion and suicidal behaviors was examined by a few review studies.^14,15^ So far, the only meta-analysis that assessed the relationship between religion and suicide is the study conducted by Wu et al in 2015.^16^ They only investigated the association between religion and suicide death. Nonetheless, the relationship between religion and other aspects of suicidal behaviors has not been fully assessed. Furthermore, several epidemiological studies addressing the relationship between religion and suicide have been performed and published on the relationship between religion and suicide since then. In light of the aforementioned issues, this meta-analysis aimed to update the results of the previous one with current evidence and assess the relationship between religion and all aspects of suicidal behaviors, such as suicidal ideations, suicide plans, suicide attempts, and suicide deaths.

## Methods

 The Vice-chancellor of Research and Technology, Hamadan University of Medical Sciences, approved and funded this systematic review. We prepared this report based on the Preferred Reporting Items for Systematic reviews and Meta-Analyses (PRISMA) statement.^17^

###  Eligibility criteria

 The exposure of interest was religious beliefs and/or practices, for example, people who manifest devotion to a deity, believe in God or gods, and follow the practices of a religion. We considered people religious regardless of what religion (Islam, Christianity, Judaism, Buddha, Hindu, Shinto, etc.) they believed in and how frequently they attended religious services. The believers were compared with nonbelievers or atheism, and the outcome of interest was suicide. Suicidal behaviors were categorized as suicidal ideation (seriously thinking about committing suicide during the past 12 months or lifetime), suicidal plan (making a plan to commit suicide during the past 12 months or lifetime), suicide attempt (actually attempting suicide during the past 12 months or lifetime), and completed suicide (suicide death).^18^ We excluded those studies addressing religiously motivated terrorism and suicidal operations.

 Observational studies (cohort, case-control, and cross-sectional studies) addressing the relationship between religion and suicidal behaviors were included regardless of language, publishing date, nationality, race, age, and gender. The studies that compared suicide rates between different religions or did not discriminate among different types of suicidal behaviors were excluded. The studies investigating the suicidal terrorist attacks were also ruled out.

###  Information sources and search

 Major electronic databases, including MEDLINE, Web of Science, and Scopus were searched for articles until 26 April 2021 using the keywords: (suicide or suicidal or suicidality) and (religion or religious or religiosity or spirituality or spiritual). The reference lists of the included papers were screened to identify more eligible studies.

###  Study selection

 The search results were combined using EndNote reference manager software, and duplicate papers of the same study were removed. The titles and abstracts of the papers were screened and ineligible studies were excluded by two authors (JP and FG) independently. Disagreements were resolved by discussion. The full text of the potentially eligible papers was retrieved and examined for further evaluation.

###  Data extraction

 The necessary extracted data from relevant studies were imported into an electronic datasheet prepared by Stata software. The following information was extracted: first author’s name, year of publication, country, study population (general population, patients with mood disorders, students, workers, veterans, as well as lesbian, gay, bisexual, and transgender (LGBQ) people), age mean/range, gender, study design (cohort, case-control, cross-sectional), suicidal behaviors (ideation, plan, attempt, death), effect estimate (risk ratio, odds ratio), sample size, effect size and its related 95% confidence intervals (CIs).

###  Methodological quality

 The Newcastle Ottawa Scale (NOS) was used for assessing the quality of the included studies.^19^ Based on this tool, each study is judged on three domains: (a) the selection of the study groups, (b) the comparability of the groups, (c) and the ascertainment of the exposure/outcome of interest. Each item of high quality is given a star. Up to nine stars were assigned to the highest-quality studies. Studies with six or fewer stars were deemed low-quality, while those with seven or more stars were regarded as high-quality.

###  Heterogeneity and reporting biases

 Heterogeneity across studies was examined by χ^2^ test,^20^ and its quantity was measured by the I^2^ statistic.^21^ Meta-regression analysis was performed to explore the sources of heterogeneity. The following variables were considered potential sources of heterogeneity: six WHO regions (Region of the Americas, European Region, Eastern Mediterranean Region, South-East Asian Region, Western Pacific Region, African Region), type of population (general population, people with mental/mood disorders, veterans, students, workers, people with comorbidities, drug disorders, LGBQ people), gender (female, male), study design (cohort, case-control, cross-sectional), suicide time (last month, last year), type of belief (just religious beliefs, religious observance), adjustment (adjusted, unadjusted), and quality of the studies (high, low). The possibility of publication bias was explored using Egger’s test,^22^ Begg’s test,^23^ and the trim-and-fill method.^24^

###  Summary measures

 The relationship between religion and suicidal behaviors was measured using risk ratio (RR) and odds ratio (OR) with their 95% CIs. Wherever reported, we used full adjusted forms of RR and OR controlled for at least one or more potential confounding factors. The data were analyzed at a significance level of 0.05 using the random-effects model.^25^ The Stata software (version 16) and RevMan (version 5.4.1) were used for data analysis.

###  Sensitivity analysis

 When between-study heterogeneity was moderate to high (I^2^ ≥ 50%), the sensitivity analysis was performed using the MetaPlot Stata command based on the sequential algorithm.^26-28^

## Results

###  Description of studies

**Figure 1:**
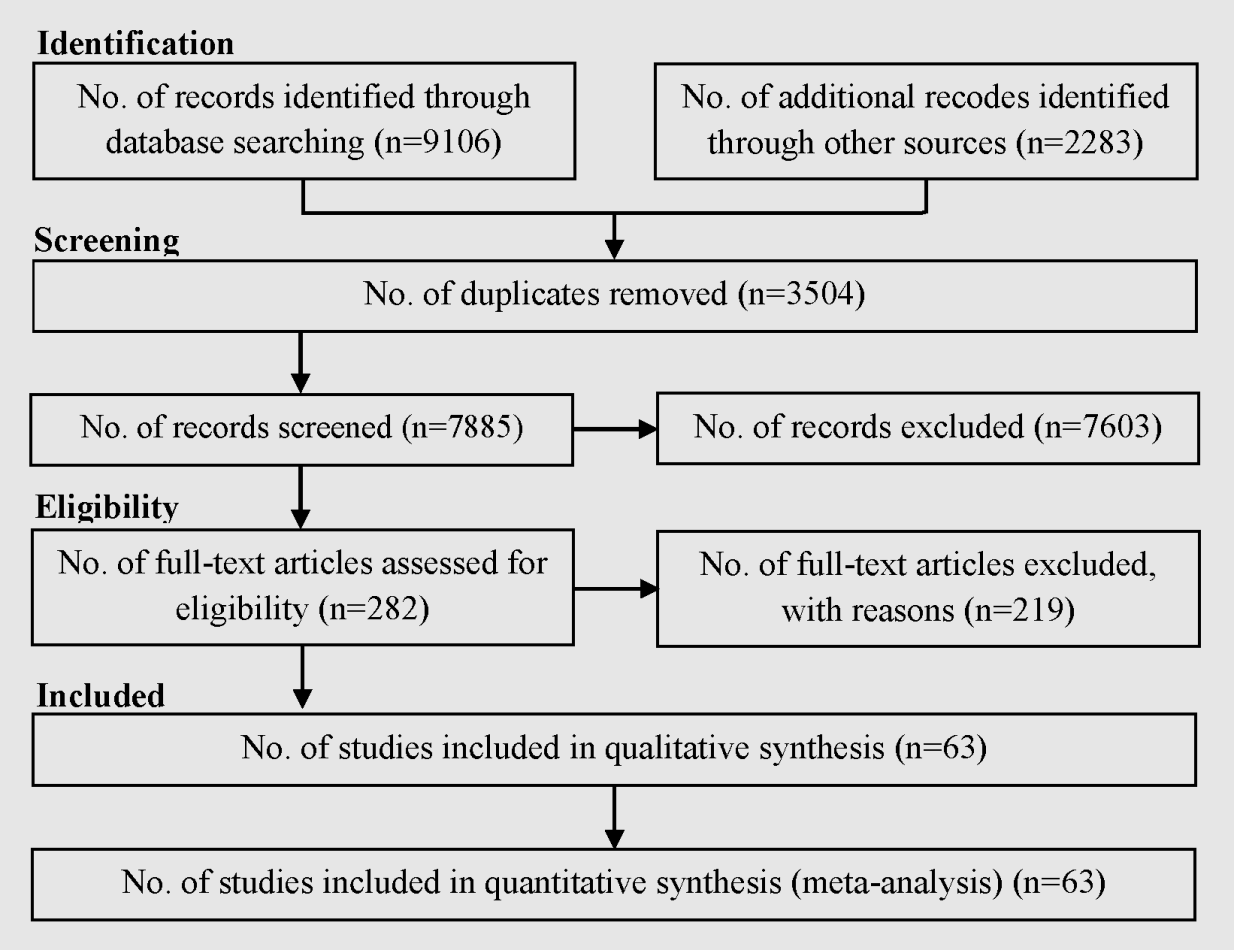
Flow of information through the different phases of the systematic review.

 A total of 11 389 references, including 9106 articles, were identified through searching the electronic databases until 26 April 2021, and 2283 articles through screening the reference list of included studies. After the removal of 3504 duplicates, 7603 references were excluded after screening their titles and abstracts. Out of 282 references considered potentially eligible after screening, 219 were excluded since they lacked one or more Population, Intervention, Comparison, Outcomes and Study (PICOS) criteria. Some papers did not separate suicidal ideation from attempted suicide cases, some reported self-harm rather than suicide, some did not report the association numerically, and some others were review articles rather than original articles. Finally, 63 references^29-91^ remained for meta-analysis (Figure 1) involving 8 053 697 participants. Based on the NOS, the quality of 49 studies was high and the quality of 14 studies was low (Table 1).

**Table 1:** Characteristics of the included studies

**1** ^st^ ** Author year**	**Country**	**Study population**	**Age**	**Sex**	**Study design**	**Religion**	**Sample**	**NOS-stars**	**Quality**
Abdu 2020	Ethiopia	Students	21.00	Both	Cross-sectional	All religions	523	*********	High
Akbari 2015	Iran	General population	25.86	Both	Case-control	All religions	600	*********	High
Almasi 2009	Hungary	General population	33-64	Both	Case-control	All religions	388	*********	High
Almeida 2012	Australia	General population	60-101	Both	Cross-sectional	All religions	21 290	*******	High
Blackmore 2008	USA	General population	32.00	Both	Cross-sectional	All religions	36 984	*******	High
Blosnich 2020	USA	LGBQ people	18.29	Both	Cross-sectional	All religions	40 150	*********	High
Brito 2021	France	General population	18-60	Both	Cross-sectional	All religions	38 694	*********	High
Burlaka 2020	Ukrain	Students	19.19	Both	Cross-sectional	Christianity	1005	*********	High
Burshtein 2016	Israel	General population	18-34	Both	Cross-sectional	Judaism	4914	*********	High
Canu 2020	Switzerland	Workers	18-65	Male	Cohort	All religions	1 534 564	*********	High
Caribé 2012	Brazil	General population	33.49	Male	Case-control	All religions	224	*********	High
Caribé 2015	Brazil	Mental disorders	42.95	Both	Cross-sectional	All religions	164	********	High
Chatters 2011	USA	General	18 +	Both	Cross-sectional	All religions	6082	*******	High
Currier 2017	USA	Veteran	28.60	Both	Cross-sectional	All religions	125	******	Low
de Sá SousaI 2020	Brazil	Student	16.40	Both	Cross-sectional	All religions	674	*********	High
Dervic 2004	USA	Mental disorders	36.80	Both	Cross-sectional	All religions	371	*****	Low
Duberstein 2004	USA	General population	68.30	Both	Case-control	All religions	172	*********	High
Fellingham 2000	USA	General population	15-34	Male	Cohort	Christianity	1 100 620	********	High
Garroutte 2003	USA	General population	33.70	Both	Cross-sectional	Christianity	1456	********	High
Hilton 2002	USA	General population	15-34	Male	Cohort	Christianity	15 555	******	Low
Hoffman 2014	USA	Students	16.04	Both	Cross-sectional	All religions	700	*********	High
Huang 2020	China	Drug abusers	39.22	Both	Cross-sectional	Buddhist	486	*******	High
Jacob 2019	Spain	General	46.30	Both	Cross-sectional	All religions	7403	*********	High
Joel Wong 2011	USA	Students	23.11	Both	Cross-sectional	All religions	1377	*******	High
Kim 2019	Korea	General population	35-49	Female	Cross-sectional	All religions	2649	******	Low
Kovess-Masfety 2011	Europe	General population	No data	Both	Cross-sectional	All religions	21 425	*********	High
Kurihara 2009	Indonesia	General population	41.40	Both	Case-control	Hindu	180	********	High
Lawrence 2016	USA	Mental disorders	No data	Both	Cross-sectional	All religions	321	*******	High
Lee 2017	Korea	General	60-90	Both	Cross-sectional	All religions	93 151	*********	High
Lester 2012	USA	General population	23.00	Both	Cross-sectional	All religions	149	****	Low
Lytle 2018	USA	LGBQ people	22.50	Both	Cross-sectional	All religions	20 702	*********	High
Martiello 2019	Italy	General population	25 +	Both	Case-control	All religions	484	******	Low
Mirzaie 2013	Iran	Students	21.16	Both	Cross-sectional	All religions	452	******	Low
Nisbet 2000	USA	General population	50 +	Both	Case-control	All religions	4863	******	Low
Nkansah-Amankra 2012	USA	General	26-34	Both	Cohort	All religions	9412	*********	High
Nonnemaker 2003	USA	Students	6-18	Both	Cross-sectional	All religions	18 924	*******	High
O'Reilly 2015	UK	General population	16-74	Both	Cohort	All religions	1 106 104	*********	High
Panczak 2013	Switzerland	General population	35-94	Both	Cohort	Christianity	3 688 617	*******	High
Peltzer 2017	Asia	Students	18-30	Both	Cohort	All religions	4675	*******	High
Rasic 2009	Canada	General population	15 +	Both	Cohort	All religions	36 984	*******	High
Rasic 2011	USA	General population	30 +	Both	Cohort	All religions	1091	*********	High
Rew 2001	USA	General population	10-19	Both	Cohort	All religions	10 059	******	Low
Robins 2009	USA	Students	18-21	Both	Cross-sectional	All religions	454	********	High
Rushing 2013	USA	Mental disorders	59 +	Both	Cross-sectional	All religions	248	*******	High
Sidhartha 2006	India	General population	12-19	Both	Cross-sectional	Hindu	1205	******	Low
Sisask 2010	Cross-National	General population	No data	Both	Case-control	All religions	8303	*********	High
Snarr 2010	USA	Veterans	No data	Both	Cross-sectional	All religions	52 780	*********	High
Stolz 2016	Multinational	General population	No data	Both	Cross-sectional	All religions	6791	*********	High
Stroppa 2013	Brazil	Mental disorders	46.20	Both	Cross-sectional	All religions	168	*****	Low
Sun 2018	China	General population	15-54	Both	Case-control	All religions	1582	*******	High
Taylor 2011	USA	General population	18 +	Both	Cross-sectional	All religions	6082	*******	High
Thanh 2006	Vietnam	General population	14 +	Both	Cross-sectional	All religions	2280	*********	High
Toussaint 2015	USA	General population	No data	Both	Cross-sectional	All religions	4448	*******	High
Trevino 2014	USA	Chronic diseases	20 +	Both	Cross-sectional	All religions	603	*********	High
Tsoh 2005	China	General population	65 +	Both	Case-control	All religions	224	*******	High
Umamaheswari 2014	India	Mental disorders	No data	Both	Case-control	Hindu	130	****	Low
Ursano 2015	USA	Veterans	18 +	Both	Cross-sectional	All religions	38 507	*********	High
VanderWeele 2016	USA	General population	30-55	Both	Cohort	All religions	89 708	*********	High
Vega Sánchez 2020	Spain	General population	No data	Both	Case-control	All religions	273	******	Low
Wang 2015	China	General population	18 +	Both	Cross-sectional	All religions	2769	********	High
Yen 2005	Taiwan	General population	65-74	Both	Cross-sectional	All religions	897	*******	High
Zhang 2010	China	General population	15-34	Both	Case-control	All religions	808	******	Low

LGBQ acronym stands for lesbian, gay, bisexual, and queer/questioning.

 The studies that addressed the association between religion and various types of suicidal behaviors were as follows: suicidal ideations (37 studies), suicide plan (3 studies), suicide attempt (32 studies), and suicide death (14 studies). The number of studies presented in the forest plots may be more than the total number of included studies since some studies reported the association between religion and different types of suicidal behaviors simultaneously. Due to substantial heterogeneity across the included studies, a meta-regression was performed considering several variables, including WHO regions, type of population, gender, study design, suicide time, type of belief, adjustment, and quality of the studies; nonetheless, neither was statistically significant.

###  Association between religion and suicide

**Figure 2:**
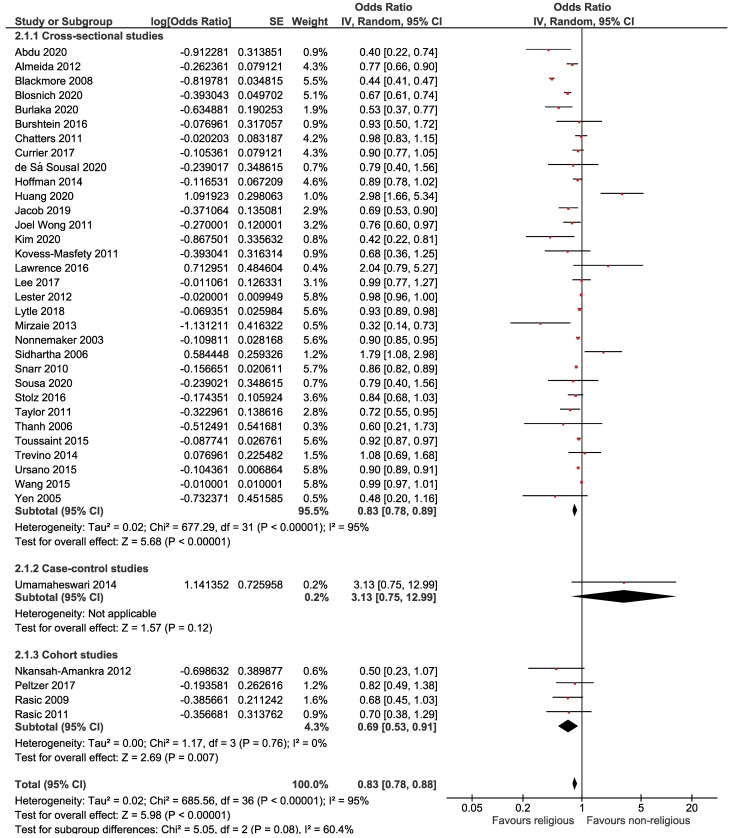
Forest plot of the association between religion and suicidal ideation.

 The association between religion and suicidal ideation is presented in Figure 2, pointing to a significant inverse association between religion and suicidal ideation. Based on this forest plot, the estimated OR of suicidal ideation for believers versus nonbelievers was 0.83 (95% CI: 0.78, 0.88). The overall effect measure demonstrated that religion significantly decreases the risk of suicidal ideation by 17% (*P* < 0.001). Between-study heterogeneity was high (I^2^ = 95%). The overall effect became slightly weaker (OR = 0.88; 95% CI, 0.84, 0.91; I^2^ = 47%) after performing a sensitivity analysis (Table 2). There was no evidence of publication bias based on the Begg test (*P* = 0.505) and Egger test (*P* = 0.130).

**Table 2:** Results of sensitivity analysis

**Variables**	**Before the sensitivity analysis**				**After the sensitivity analysis**			
	**Studies**	**χ** ^ 2 ^	**I** ^ 2 ^	**OR (95% CI)**	**Studies**	**χ** ^ 2 ^	**I** ^ 2 ^	**OR (95% CI)**
Suicidal ideation	37	0.001	95%	0.83 (0.78, 0.88)	30	0.002	47%	0.88 (0.84, 0.91)
Suicidal plan	3	0.002	84%	0.93 (0.83, 1.04)	2	0.531	0%	0.89 (0.83, 0.94)
Suicide attempt	31	0.001	86%	0.84 (0.79, 0.89)	24	0.009	45%	0.91 (0.88, 0.95)
Completed suicide	8	0.001	92%	0.31 (0.14, 0.72)	7	0.900	0%	0.25 (0.19, 0.33)

**Figure 3:**
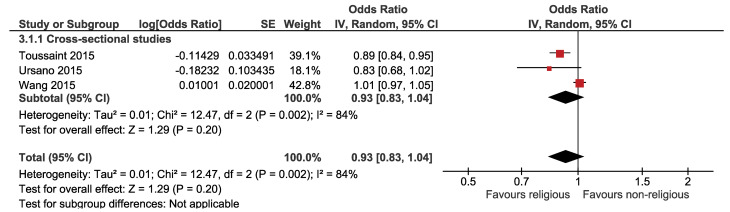
Forest plot of the association between religion and suicide plan.

 The association between religion and suicide plan is displayed in Figure 3, which illustrates that the association between religion and the suicidal plan was not statistically significant. Based on this forest plot, the estimated OR of the suicidal plan for believers versus nonbelievers was 0.93 (95% CI: 0.83, 1.04). The overall effect measure indicated that religion decreases the risk of the suicide plan by 7% (*P* = 0.200). Between-study heterogeneity was high (I^2^ = 84%). The overall effect became stronger and significant (OR, 0.89; 95% CI, 0.83, 0.94; I^2^ = 0%) after performing a sensitivity analysis (Table 2). There was no evidence of publication bias based on the Begg test (*P* = 0.602) and Egger test (*P* = 0.445).

**Figure 4:**
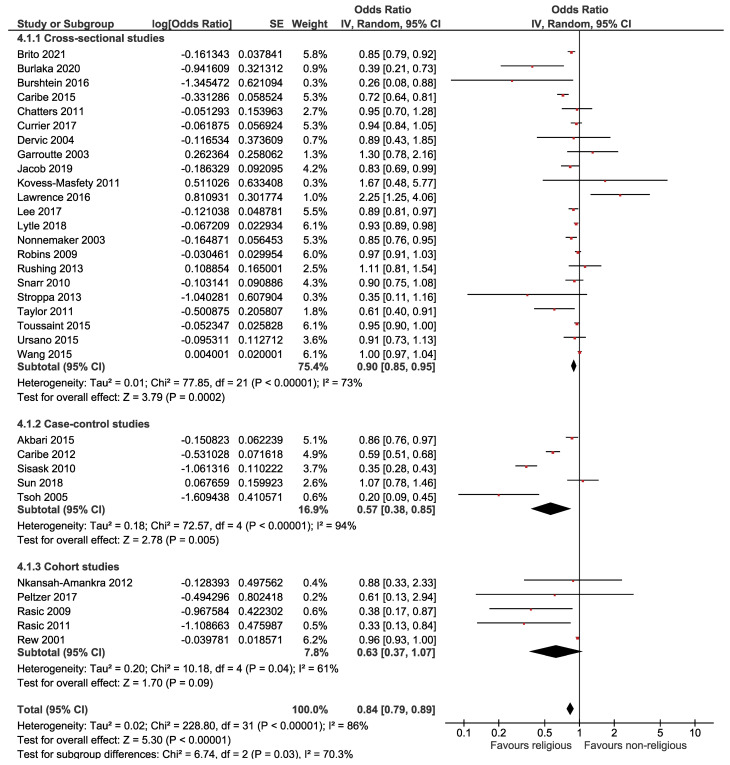
Forest plot of the association between religion and suicide attempt.

 The relationship between religion and suicide attempts is displayed in Figure 4, revealing a significant inverse association between religion and suicide attempts. Based on this forest plot, the estimated OR of suicide attempts for believers versus nonbelievers was 0.84 (95% CI: 0.79, 0.89). The overall effect measure shows that religion decreases the risk of the suicidal plan by 16% (*P* < 0.001). Between-study heterogeneity was high (I^2^ = 86%). The overall effect became weaker (OR = 0.91; 95% CI, 0.88, 0.95; I^2^ = 45%) after performing a sensitivity analysis (Table 2). The Begg test revealed no evidence of publication bias (*P* = 0.347); however, the Egger test did show evidence of publication bias (*P* = 0.007). However, the trim-and-fill analysis estimated no missing studies.

**Figure 5:**
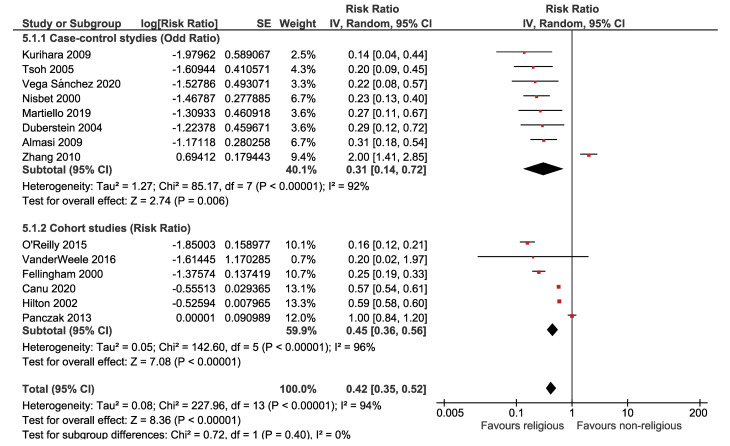
Forest plot of the association between religion and suicide death.

 The association between religion and suicide death is presented in Figure 5, which shows a significant inverse association between religion and suicide death. According to the forest plot, the estimated OR of suicide attempts for believers versus nonbelievers was 0.31 (95% CI: 0.14, 0.72). The overall effect measure demonstrates that religion decreases the risk of suicide death by 69% (*P* < 0.001). Between-study heterogeneity was high (I^2^ = 92%). The overall effect became stronger (OR, 0.25; 95% CI, 0.19, 0.33; I^2^ = 0%) after performing a sensitivity analysis (Table 2). There was no evidence of publication bias based on the Begg test (*P* = 0.928) and Egger test (*P* = 0.177).

## Discussion

 The results of this meta-analysis pointed to the overall mild-to-moderate protective relationship of religiosity with suicidal ideation, suicide plans, and suicide attempt, as well as a strong protective relationship between religiosity and suicide death. Nevertheless, the observed association between religion and suicidal behaviors does not necessarily imply a direct cause-and-effect relationship. Suicide is a highly complex issue that is associated with a range of risk and protective factors at both individual and contextual levels.^3,6,7^ Religion is a multi-factorial phenomenon; therefore, we cannot regard risk and protective factors as individual items, rather they should be deemed as a cluster. Diseases are promoted by risk factors while being prevented by protective factors. In this regard, diseases will not develop if risk and protective factors are in balance or if protective factors dominate risk factors.^92^ Therefore, the role of religion in the prevention of suicide should be considered, along with other influential factors.

 A vast majority of literature observes a protective effect of religion on suicidal behaviors rather than supports. Several mechanisms have been proposed to explain the protective role of religiosity against suicide. Most religions have strict prohibitions against suicide; therefore, those who are more committed to such religions are less likely to commit suicide. Furthermore, it has been proposed that all major religions discourage all forms of violence, including suicide, and advocate peace and unity which may be deemed life-affirming values, thereby preventing suicide.^15,93^ In addition to sanctioning suicide, participation in organized religions allows members of the congregation and clergies to form an extended support network, which has been demonstrated to be a protective factor against suicidal behaviors.^94^ Religious belief has also been linked to lower levels of violence and hostility which have constantly been shown to be associated with suicidal behaviors.^95^ Furthermore, many religions forbid illegal activities, including substance misuse, alcohol consumption, and smoking which have been associated with suicide.^8-10^ Therefore, the restriction of high-risk behaviors may have an indirect protective impact against suicide.

 There was considerable heterogeneity across the included studies (small *P* value of χ^2^ and large I^2^ statistic). The results of the statistical tests used to examine heterogeneity should be interpreted cautiously. The χ^2^test has low statistical power when the sample size is small or the number of studies is limited. The test, on the other hand, has high power in detecting a modest level of heterogeneity when the sample size or number of the included studies is large.^20^ Consequently, a portion of the observed heterogeneity can be attributed to the large sample size (involving 8 053 697 participants) and the great number of studies included in the meta-analysis. Nevertheless, inconsistencies across studies can account for a portion of the observed heterogeneity. The observed heterogeneity can be justified on the ground that the results of individual studies come from varied settings with different religions, as well as varying degrees of religious fidelity and adherence to religious teachings. This diversity may be a source of the observed heterogeneity.

 Wu et al^16^ conducted a meta-analysis in 2015 to examine the association between religion and completed suicide. They found nine studies that altogether included 2339 suicide cases and 5252 participants. They reported an overall protective effect of religiosity from completed suicide (OR = 0.38; 95% CI: 0.21, 0.71) and concluded that religion may play a protective role against suicide in a majority of settings. The results of the referred research were consistent with the findings of the present study. The overall measure produced from OR, estimating the probability of completed suicide, was larger than that obtained from RR, as depicted in Figure 4. The rationale for this is straightforward since OR tends to overstate the degree of the relationship.^96^

 This meta-analysis is associated with a few limitations and considerations that should be taken into account when interpreting the results. Firstly, the studies included in this meta-analysis, except in a few cases, did not set out to assess the effect of different types of religions, denominations, intensity, and spirituality on suicidal behaviors. Therefore, religion was treated as a binary entity and neither captured this dimensionality nor measured the effect of different aspects of religion on suicidal behaviors. Secondly, the number of studies addressing the association between religion and “suicide plans” was relatively small. This issue reduced the strength of association and the generalizability of the results considering the relationship between religion and suicide plans. Thirdly, we imported the adjusted forms of RR and OR into the meta-analysis wherever feasible. Nevertheless, the confounding effect could not be entirely ruled out since some studies provided crude forms of RR or OR estimates. This problem might lead to an overestimation of the overall effect size of religion. Fourthly, there were eight studies (mainly old studies) that appeared to be eligible for this meta-analysis; nonetheless, their full texts were not available and their corresponding authors did not respond. This issue might raise the possibility of selection bias. Finally, we did not evaluate the religiously motivated suicidal operations and terrorism which is a matter of a completely different nature and has not been the subject of this research. Despite the aforementioned limitations, we developed a wide search strategy to include as many studies as possible, including 56 studies involving 8 053 697 participants. The current meta-analysis was able to examine the association between religiosity and the overall suicide burden.

## Conclusion

 This meta-analysis addressed the association between religiosity and suicide. The results of this study support the notion that religion can play a protective role against suicidal behaviors. Based on current evidence, religious affiliation and participation significantly decreased the risk of suicidal ideation, suicide plans, suicide attempt, and completed suicide. Although this association does not necessarily imply causation, an awareness of the relationship between religion and suicide risk can be of great help in suicide prevention policies and procedures.

## Acknowledgments

 We would like to appreciate the Vice-Chancellor for Research and Technology of the Hamadan University of Medical Sciences for approval of this study.

## Authors’ contribution

 Jalal Poorolajal contributed to the study conception and design, analysis and interpretation of data, and drafting of the manuscript. Mahmoud Goudarzi contributed to the study design and critical revision. Fatemeh Gohariensaf contributed to the acquisition of data and critical revision. Nahid Darvishi contributed to the study design, acquisition of data, and critical revision. All authors approved the final manuscript as submitted and agree to be accountable for all aspects of the work.

## Availability of data and material

 Not available.

## Conflicts of interest

 The authors declare that they have no conflict of interest.

## Consent for publication

 All authors agree with the publication of this manuscript in the current format.

## Ethics approval

 There was no human subject in this study.

## Funding

 The Vice-Chancellor of Research and Technology, Hamadan University of Medical Sciences funded this study (140006164895). However, the funder had no role in the study design, data collection, and analysis, decision to publish, or manuscript preparation.

**Highlights**

- This meta-analysis revealed the extent to which religion can affect suicidal behaviors (i.e., ideation, plan, attempt, and death).
- This meta-analysis pointed to the inverse association of religion with suicidal ideation, suicidal plan, suicide attempt, and suicide death.
- Religion reduced the risk of suicidal ideation, suicidal plan, suicide attempt, and suicide death by 17%, 7%, 16%, and 69%, respectively.
- The results of this meta-analysis can be of great help in designing suicide prevention policies and programs.
